# Vitamin D-Mediated Anti-cancer Activity Involves Iron Homeostatic Balance Disruption and Oxidative Stress Induction in Breast Cancer

**DOI:** 10.3389/fcell.2021.766978

**Published:** 2021-11-08

**Authors:** Khuloud Bajbouj, Lina Sahnoon, Jasmin Shafarin, Abeer Al-Ali, Jibran Sualeh Muhammad, Asima Karim, Salman Y. Guraya, Mawieh Hamad

**Affiliations:** ^1^College of Medicine, University of Sharjah, Sharjah, United Arab Emirates; ^2^Research Institute for Medical and Health Sciences, University of Sharjah, Sharjah, United Arab Emirates; ^3^College of Health Sciences, University of Sharjah, Sharjah, United Arab Emirates

**Keywords:** vitamin D, breast cancer, iron, oxidative stress, cell death

## Abstract

**Background:** Vitamin D deficiency associates with high risk of breast cancer (BRCA) and increased cellular iron. Vitamin D exerts some of its anti-cancer effects by regulating the expression of key iron regulatory genes (IRGs). The association between vitamin D and cellular iron content in BRCA remains ambiguous. Herein, we addressed whether vitamin D signaling exerts a role in cellular iron homeostasis thereby affecting survival of breast cancer cells.

**Methods:** Expression profile of IRGs in vitamin D-treated breast cancer cells was analyzed using publicly available transcriptomic datasets. After treatment of BRCA cell lines MCF-7 and MDA-MB-231 with the active form of vitamin D, labile iron content, IRGs protein levels, oxidative stress, and cell survival were evaluated.

**Results:** Bioinformatics analysis revealed several IRGs as well as cellular stress relates genes were differentially expressed in BRCA cells. Vitamin D treatment resulted in cellular iron depletion and differentially affected the expression of key IRGs protein levels. Vitamin D treatment exerted oxidative stress induction and alteration in the cellular redox balance by increasing the synthesis of key stress-related markers. Collectively, these effects resulted in a significant decrease in BRCA cell survival.

**Conclusion:** These findings suggest that vitamin D disrupts cellular iron homeostasis leading to oxidative stress induction and cell death.

## Introduction

It is well-established that potent forms of vitamin D, mainly 1,25-dihydroxycholecalciferol, play important roles in human development and physiology, especially in bone metabolism and the regulation of calcium and phosphorus levels. Moreover, numerous studies elaborated that vitamin D has a role in the development, progression, and treatment of numerous disease states including cardiovascular diseases, autoimmunity, and cancer (Jeon and Shin, 2018). Previous work showed that deficiency of vitamin D is highly associated with a high risk of breast cancer (BRCA) development (Eliassen et al., 2016; Iqbal and Khan, 2017) as it has anti-neoplastic effects against the disease (Eliassen et al., 2016). It was previously reported that there is a strong negative correlation between vitamin D levels and BRCA incidence in postmenopausal women (Abbas et al., 2009; Edlich et al., 2009). Moreover, patients with BRCA were shown to have lower vitamin D levels relative to healthy individuals (Hatse et al., 2012). When the level of vitamin D in BRCA patients is < 20 ng/mL, it is often associated with poor prognosis, metastasis (>90% of cases), and death (>70% of cases) (Imtiaz et al., 2012). Vitamin D and its mechanism of action as an anti-cancer agent were supported by different studies (Honma et al., 1983; Krishnan et al., 2012; Miseta et al., 2015). For example, it was reported that vitamin D can upregulate the expression of p21WAF 1/Cip 1 and p27Kip 1 which are the cell cycle regulators thus limiting the proliferative potential of cancer cells (Gartel and Tyner, 2002; Audo et al., 2003). It was also reported to prompt retinoblastoma (Rb) protein dephosphorylation leading to cell cycle arrest in multiple cancer cells *in vitro* (Audo et al., 2003). *In vitro* studies stated that vitamin D has the ability of BRCA cell lines inhibition as well as induction of apoptosis by upregulating the expression of p21WAF1/Cip, p53, and Bax (caspase activator) genes and by reducing the expression of Bcl-2 (anti-apoptotic mediator) (Calvert et al., 1998; Gartel and Tyner, 2002; Audo et al., 2003).

Iron is an essential nutrient that is intricately involved in the various metabolic processes including cellular respiration and bioenergetics and cell growth and proliferation among others (Winterbourn, 1995). Although iron availability is essential for cells and organisms, excess iron could result in the production and propagation of reactive oxygen species (ROS) by Fenton chemistry (Dixon et al., 2012; Miseta et al., 2015). That would lead to cell damage, generation of oxidative stress, and death by apoptosis and ferroptosis (Mancias et al., 2014; Yang et al., 2014; Hou et al., 2016). Increased cellular iron content in BRCA has been previously reported to associate with poor prognosis and chemoresistance (Habashy et al., 2010). Differential expression of key iron regulatory genes (IRGs) including HAMP, ferroportin (FPN; SLC40A1), and transferrin receptor 1 (TFRC; CD71) has been widely reported as significant biomarkers in BRCA (Pinnix et al., 2010). For example, reduction in the expression of FPN, high ferritin, hepcidin, transferrin receptor (TfR1; CD71) expression, and high labile iron content (LIP) are consistent with the findings associated with breast cancer (Habashy et al., 2010; Pinnix et al., 2010).

Vitamin D was previously reported to reverse iron-mediated oxidative stress (Uberti et al., 2016) and to differentially alter HAMP expression in a tumor type-specific manner (Welsh et al., 2002). Vitamin D treatment in hepatocellular carcinoma was reported to reduce hepcidin and ferritin production and increase in FPN (Bacchetta et al., 2014). Additionally, previous reports have indicated that vitamin D can exert some of its anti-cancer effects by regulating the IRGs expression (Zughaier et al., 2014), suggesting that the treatment effectiveness of BRCA cellular iron metabolism with vitamin D remains ambiguous. In this context, previous work also suggested that vitamin D could exert an anti-growth effect on BRCA cells by modulating several metabolic pathways (Swami et al., 2003). Additionally, preliminary *in silico* data seems to suggest that treatment with calcitriol resulted in a significant decrease in the viability of BRCA cells. In relation to these findings, we have hypothesized that treatment with vitamin D may enhance cell death in BRCA cells by reducing cellular iron content. To address this issue, labile iron content, expression of IRGs at the protein level, oxidative stress, and cell survival were evaluated in both MCF-7 and MDA-MB-231 cells post-vitamin D treatment.

## Materials and Methods

### Bioinformatics Analysis of Publicly Available Transcriptomic Data Resources

We searched for transcriptomic datasets available from National Center for Biotechnology Information (NCBI0 Gene Expression Omnibus (GEO),^1^ which is a free public genomics database. The search terms Vitamin D/Cholecalciferol/Calcitriol and breast cancer cells were used to identify datasets for human breast cancer cell lines treated with vitamin D. We selected the dataset GSE53975, which included MCF-7 and MDA-MB-231 cells treated with calcitriol or DMSO (*n* = 3 for each group; *n* = 6 per cell line and *n* = 12 in total) using the Affymetrix Human Exon 1.0 ST platform. The gene expression profiles were downloaded, and the raw data were processed using R statistical software (version 3.5.1). According to the expression profiling data, relative mRNA expression in calcitriol-treated samples vs. DMSO-treated controls was identified using the Limma package (available at http://www.bioconductor.org/packages/release/bioc/html/limma.html) in Bioconductor package version 1.0.2. A log2 fold-change (log2FC) was calculated to present differentially expressed genes (DEGs) and an adjusted *p*-value < 0.05 using classical *t*-test was applied. Gene set enrichment analysis was performed using several ontologies resources on DEGs to identify the underlying pathways using pathfinder tool. Gene ontology (GO) terms with a *p*-value < 0.01, a minimum count of 3, and an enrichment factor > 1.5 (the enrichment factor is the ratio between the observed counts and the counts expected by chance) were collected and grouped into clusters based on their membership similarities.

### Cells and Treatment Protocols

In this study, both MCF-7 and MDA-MB-231 human BRCA cell lines were used from American Type Culture Collection (Manassas, VA, United States). Dulbecco’s Modified Eagle’s Medium (DMEM) was used to maintain the cells at 37°C and 5% CO_2_ as it was supplemented with 4 mM glutamine, 2 μg/mL insulin, 10% fetal calf serum, 1 mM of sodium pyruvate, 1 mM of non-essential amino acids, and penicillin/streptomycin antibiotics. Cells at 0.5–1 × 105 cells/mL were seeded using 25 cm flasks and the treatment with different concentrations of calcitriol (25 hydroxyvitamin D; the active form of vitamin D) from (Abcam) was performed at a confluency of ∼70% for different time points. Control cells were left untreated or treated with DMSO.

### Calcitriol Treatment and MTT Cell Viability Assay

Cell viability was determined in cells treated with vitamin by using a colorimetric assay MTT (3-(4,5-dimethylthiazol-2-yl)-2,5-diphenyltetrazolium bromide (Sigma-Aldrich), 104 cells of vitamin D treated and untreated control cells were grown in 96-well plates with 0.2 mL culture media and were cultured for 24, 48, and 72 h. After that MTT salt was added and mixed with the cells then kept for 2 h incubation in a humidified incubator at 37°C and 5% CO_2_. Product of MTT formazan was dissolved in DMSO and then reading of absorbance was taken at 570 nm using a microplate reader.

### Quantitative Real-Time PCR

The cDNA was synthesized from 1 μg of total RNA using the QuantiTect Reverse Transcription Kit (Qiagen) according to the manufacturer’s protocol. qPCR was performed using 1:l of complementary DNA (cDNA), specific primers for each genes as listed, SYBR^®^ Green I, and an iCycler Thermal Cycler. Expression levels of target human genes: hepcidin gene expression were (forward: 5′-CTGTTTTCCCACAACAGACG-3′, reverse: 5′-CAGCACATCCCACACTTTGA-3′) and ferroportin (forward: 5′-CAGTTAACCAACATCTTAGC-3′, reverse: 5′-AAGCTCATGGATGTTAGAG-3′) were normalized to GAPDH expression (forward: 5′-CCAGGTGGTCTCCTCTGACTTC-3′, reverse: 5′-TCATACCCAGGAAATGAGCTTGACA-3′).

### Western Blot Analysis

Post-vitamin D treatment both MCF-7 and MDA-MB-231 cells were collected and lysed using NP-40 lysis buffer (1.0% NP-40, 150 mM of NaCl, 50 mM of Tris–Cl, pH 8.0) containing protease cocktail inhibitor tablets (Sigma, Germany). Protein quantification was performed using the standard Bradford method. After that protein lysates were separated on 12% sodium dodecyl sulfate–polyacrylamide gel electrophoresis (SDS-PAGE), proteins were transferred into nitrocellulose membrane using semi-dray and/or wet transfer method. Membranes were blocked in 5% skimmed milk for 1 h at room temperature. Proteins of interests were detected using the following different types of primary monoclonal antibodies (anti-TfR1, anti-TfR2, anti-hepcidin, anti-survivin, and anti-HIf1-α all from Abcam, United Kingdom, anti-γH2AX from Millipore, Billerica, MA, United States, and anti-FTH1 from LSBio, United States) diluted at 1:1,000 and kept overnight at 4°C on the shaker. For oxidative stress enzymes, we used anti-HO-1 (Thermo Fisher Scientific, United States), anti-catalase (Abcam, United Kingdom). Secondary labeled anti-mouse and anti-rabbit antibodies from (Cell Signaling) were reacted with the membrane for 1 h at room temperature. Detection of chemiluminescence was performed using an ECL kit (Bio-Rad, United States). Bio-Rad Image Lab software (ChemiDoc^TM^ Touch Gel and Western Blot Imaging System; Bio-Rad) was used to quantify protein bands and β-actin was used as the normalization control. Control sample values were defined as 1.00 and the protein level changes of treated samples were quantified relative to the control.

### Labile Iron Measurement

Labile iron content was measured and assessed as it was previously described but a slight modification was performed where deferoxamine was used as a chelator instead of deferiprone (Prus and Fibach, 2008). In brief, cells were washed two times with PBS then 0.5 × 106 was incubated with 0.125 μM calceinacetoxymethyl ester (Cat. No. 56496, Sigma Aldrich) for 15 min at 37°C. Two times washing was performed, and cells were kept for 15 min incubation with 100 mM DFO. Flow cytometry analysis was performed (The BD FACSAria^TM^ III, Becton-Dickinson) applying a 488-nm laser beam for excitation. As a minimum number of events was 50,000 that were collected/sample and percentage of positively stained cells was computed to the 99% level of confidence. Mean fluorescence intensity (MIF) was presented and as it represents the geometric mean fluorescence intensity of a log-normal distribution of fluorescence signals. MFI level increases as the content of free iron decrease; ΔMFI is a qualitative measurement of LIP changes (MFICA-AM/DFO-MFICA-AM alone). If ΔMFI > 0, this would indicate LIP availability while ΔMFI <0 indicates depletion of LIP.

### Reactive Oxygen Species Measurement

Antioxidant related enzymes were measured using the ROS-Glo H_2_O_2_ Assay Kit (Promega, Cat No. G8820, United States) in both MDMBA-321 and MCF-7 cells, 5,500 cells/well were seeded using a 96-well plate and treated with calcitriol substrate as per manufacturer’s instructions to assess ROS production for 3 and 6 h; cells that were plated and left untreated before ROS quantification served as controls.

### Proteome Profiler Array

Human Cell Stress Array Kit (R&D system, Cat No. ARY018, United States) was used to detect 26 different cell stress related proteins in both cell lines. After protein quantification by using the standard Bradford method, four nitrocellulose membranes, each containing 26 different capture antibodies, were blocked for 1 h by Array Buffer 6 at room temperature on a shaker. Cell lysate that contains 300 μg of protein was prepared with Array Buffer 4 and 20 μL of detection antibody cocktail. Samples were loaded onto the membrane overnight at 2–8°C. Chemiluminescence was detected by Streptavidin-HRP methods using the dilution factor suggested by the manufacturer. A Bio-Rad Image Lab software (ChemiDoc^TM^ Touch Gel and Western Blot Imaging System; Bio-Rad) was used for protein dot quantification. Reference spots were used as a normalization control and control sample values were defined as 1.00 and the values of treated samples were quantified relative to the control.

### Cell Cycle Progression Analysis

Analysis of a cell cycle progression was performed by using Propidium Iodide Flow Cytometry Kit (Abcam, United Kingdom). Briefly, cells were seeded at a density of 1 × 106 cells/mL. After indicated treatment time points, cells were harvested and washed with PBS, cells were then resuspended in 0.5 mL ice-cold PBS and fixed for 48 h with 4 mL of ice-cold 70% ethanol at −20°C. The cell pellet was washed again twice with ice-cold PBS, resuspended, and incubated with 0.2 mL of staining buffer that is supplemented with stain solution containing RNase and propidium iodide (PI) at room temperature in the dark. Phases of the cell cycle with different DNA contents were determined by performing flow cytometry (The BD FACSAria^TM^ III; Becton Dickinson and Company). The cell cycle platform of the FlowJo software was used to analyze the distribution of the cell cycle and percentage of cells in sub-G1, G1, S, and G2/M phases in addition to the Watson pragmatic model (Tree Star).

### Annexin-V Staining for Apoptosis Detection

Cell apoptosis was performed using Annexin V-FITC Apoptosis Staining/Detection Kit protocol from (Abcam, United States). In brief, 1 × 106 cells/mL were seeded, treated, and harvested then washed with PBS two times. Cells were kept for 20 min with 0.2 mL staining buffer annexin-V/PI in the dark at room temperature. Cell apoptosis was analyzed using flow cytometry (The BD FACSAria III; Becton Dickinson and Company) with an excitation wavelength of 488 nm; a 530/30 nm band pass filter for fluorescein detection and a long pass filter of 670 nm was used. Apoptotic and necrotic cells were analyzed as the following: PI only positive cells were counted and indicated necrosis, cells which were counted as annexin V positive and PI negative were indicated as early apoptotic cells, and cells which were counted as annexin V positive and PI positive were indicated as late apoptotic cells. FlowJo software with the Watson pragmatic model (Tree Star, Ashland OR, United States) was used for data analysis of the flow results.

### Statistical Analysis

Graph prism Pad 5 software (GraphPad Software Inc., La Jolla, CA, United States) was used for data analysis of cell viability and iron regulation. Other data paired *t*-test was used for *p*-values generation to compare between groups in each data set.

## Results

### Subsection

#### Vitamin D Differentially Alters the Expression Profile of Iron Regulatory Genes

**FIGURE 1:**
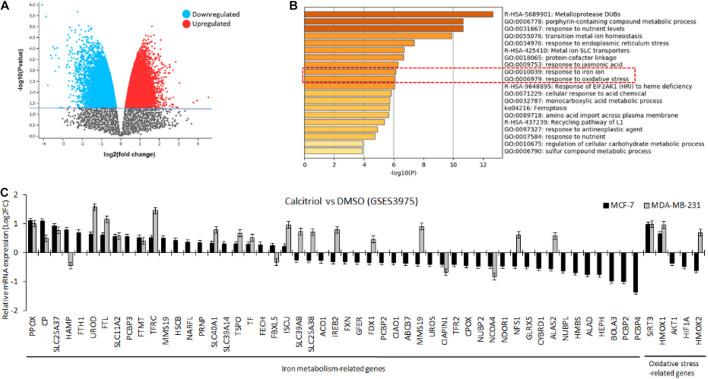
*In silico* analysis to investigate the effect of vitamin D treatment on breast cancer cells. **(A)** Volcano plot showing more than 5,000 genes were significantly up-/downregulated in MCF-7 and MDA-MB-231 cells. **(B)** Pathway analysis of differentially expressed genes showing enrichment of pathways related to the response to iron ion and oxidative stress. **(C)** Bar graph plot showing the mean expression values of iron metabolism and oxidative stress-related genes for both cell lines treated with calcitriol compared with DMSO-treated cells.

To investigate the effect of vitamin D on breast cancer cells, we first performed *in silico* analysis of a publicly available expression array dataset of breast cancer cells treated with 40–50 μM of calcitriol. Both breast cancer cell types, MCF-7, and MDA-MB-231, showed more than 5000 DEGs (Figure 1A, significantly up/downregulated, *p* < 0.05). Disruption of iron metabolism and induction of oxidative stress is known to induce a deleterious effect on cancer cells. Therefore, we aimed to explore if the DEGs in vitamin D-treated breast cancer cells included iron metabolism-related and oxidative stress-related genes. Utilizing the DEGs common to both MCF-7 and MDA-MB-231 cell lines treated with vitamin D, we performed pathway enrichment analysis. The results showed a significant enrichment of GO pathways related to response to iron ion and response to oxidative stress (Figure 1B, GO:001039, GO:0006979, *p* < 0.01). Other significantly enriched genes set in these treated breast cancer cells included metalloproteases, porphyrin-containing compound metabolic process, and response to nutrient levels.

Next, the mean expression values were plotted of those genes for both cell lines treated with calcitriol compared with DMSO-treated cells. We found that 56 genes related to iron metabolism and oxidative stress were significantly dysregulated in MCF-7 cells but only 26 of those genes were dysregulated in MDA-MB-231 cells (Figure 1C). Few of the critical IRGs, such as CP, FTH1, SLC11A2, SLC40A1, and SLC39A14, were upregulated, and TFR2 was downregulated upon calcitriol treatment in both types of cell lines. Additionally, SIRT3 and HMOX, key oxidative stress markers, were also found upregulated in both calcitriol-treated cell lines. Nevertheless, only a few other key IRGs, such as HAMP and FTL, were upregulated only in calcitriol-treated MCF-7 cells. This suggested us to further investigate the protein expression of few of these iron metabolism and oxidative stress on cancer cell survival in vitamin D-treated breast cancer cells.

#### Vitamin D Reduces Breast Cancer Cell Viability

**FIGURE 2:**
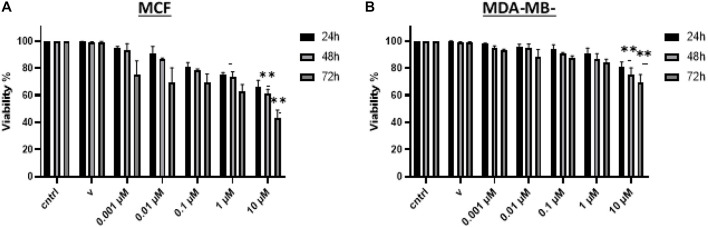
Assessment of BRCA cell viability following vitamin D treatment. Percentage cell viability of **(A)** MCF-7 and **(B)** MDA-MB-231 cells treated with 0.001, 0.01, 0.1, 1, and 10 μM vitamin D for 24, 48, and 72 h as measured by the MTT assay. **Represents statistically significant change (*P* < 0.01) in cell survival between calcitriol-treated and untreated controls at each time point tested.

BRCA cell viability was examined post-treatment to test the effect of vitamin D in both MCF-7 and MDA-MB-231 by using the MTT assay. In Figure 2, it is shown that cell viability was significantly reduced in cultures that were treated with 0.001, 0.01, 0.1, 1, and 10 μM of vitamin D for 24, 48, and 72 h; this was true for MCF-7 and MDA-MB-231 cells. There is a significant reduction in cell viability at 24 (72%), 48 (65%), and 72 (50%) h treatment of MCF-7 cells with 10 μM vitamin D as is shown in Figure 2A. There was more pronounced reduction in MDA-MB-231 cell viability when treated with 10 μM vitamin D for 72 h (79%; Figure 2B).

#### Vitamin D Differentially Disrupts Cellular Iron Metabolism in Breast Cancer Cells

**FIGURE 3:**
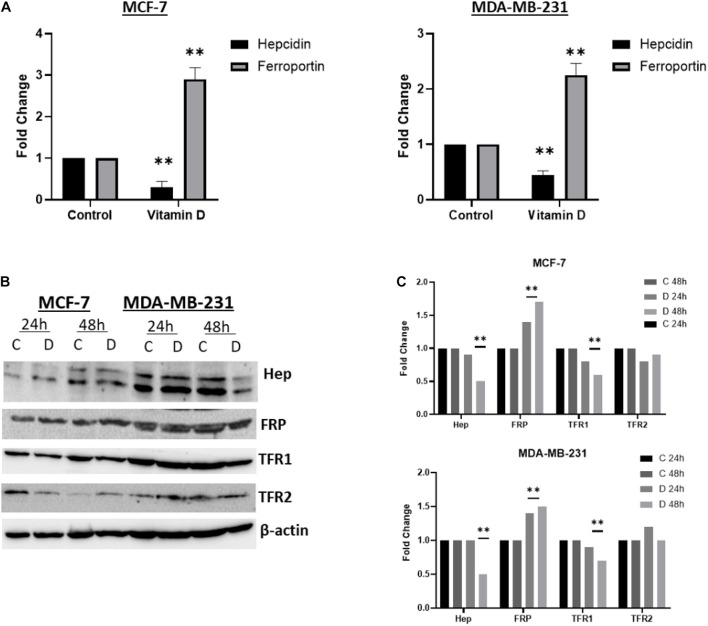
Vitamin D disrupts cellular iron metabolism in BRCA cells. The status of hepcidin, FPN, TfR1, and TfR2 in vitamin D treated MCF-7 and MDA-MB-231 cells. **(A)** Gene expression was analyzed in from MCF-7 and MDA-MB-231 cells treated with vitamin D 10 μM and cultured for 6 h. **(B)** Cell lysates were prepared from MCF-7 and MDA-MB-231 cells treated with vitamin D 10 μM and cultured for 24 and 48 h. Shown data are representative of three separate experiments. **(C)** Quantitative analysis of relative protein band density after normalization to β-actin and compared to the control. **Represents statistically significant change (*P* < 0.01).

**FIGURE 4:**
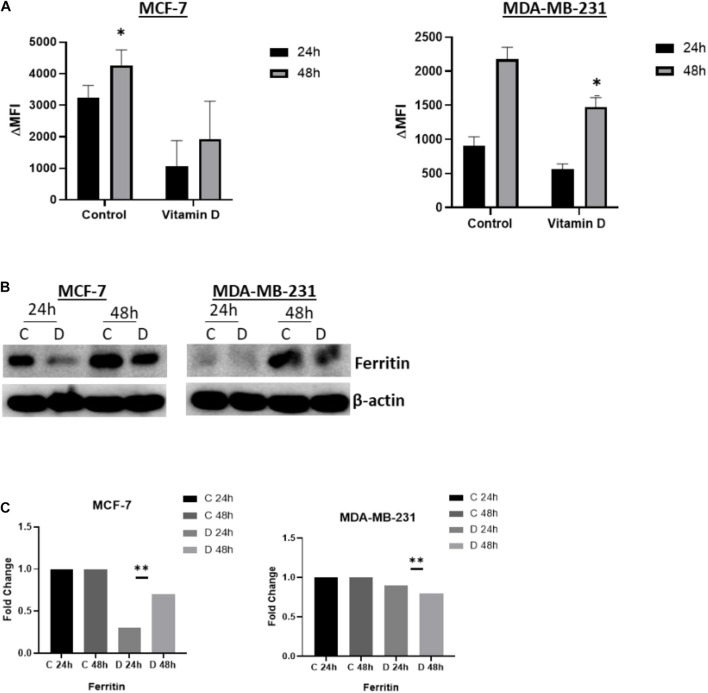
Vitamin D depletes labile cellular iron in BRCA cells. **(A)** Calcein-based flow cytometry method was used to assess labile iron pool (LIP) content in MCF-7 and MD-MBA-231 cells treated with 10 μM vitamin D. Difference in mean fluorescence intensity (ΔMFI) between calcein + chelator and calcein only is an indirect measurement of LIP content; the smaller the ΔMFI value, the lower the LIP content. **(B)** Ferritin expression was assessed in cell lysates of MCF-7 and MDA-MB-231 cells at 24 and 48 h post vitamin D treatment; untreated cells served as controls. The data shown are the mean MFI ± SEM of four separate experiments. **(C)** Quantitative analysis of relative protein band density after normalization to β-actin and compared to the control. *Represents statistically significant change (*P* < 0.05) and **Represents statistically significant change (*P* < 0.01).

A significant depletion of cellular iron was observed in the treatment of MCF-7 and MDA-MB-231 with 10 μM vitamin D and it differentially affected key IRGs expression. After calcitriol treatment, both MCF-7 and MDA-MD-231 cells showed reduction in hepcidin gene expression and protein levels (Figures 3A,B), and there was a significant increase of FPN levels in both cell types (Figures 3A,B). Additionally, in MCF-7 cells TFR1 expression showed a drop at 24 and 48 h post-treatment and in MDA-MB-231 cells at 48 h (Figure 3B). Changes were not significant in TFR2 expression following vitamin D treatment (Figure 3B). LIP content was tested in both MCF-7 and MDA-MB-231 cells following treatment with 10 μM vitamin D for 24 and 48 h to test if the reduction in hepcidin and increase in FPN would result in increased cellular iron efflux. As shown in Figure 4A, depletion of LIP (ΔMFI < 0) was evident in both cell types post-treatment with vitamin D; MCF-7 at 24 h and MDA-MB-231 at 48 h. Lysates were obtained from control and treated cells to evaluate ferritin expression. As shown in Figures 4B,C, significant changes in ferritin protein synthesis were observed in MCF-7 following vitamin D treatment at 24 h; no clear change in ferritin synthesis was evident in vitamin D-treated MDA-MB-231 cells at 24 or 48 h post-treatment.

#### Vitamin D Induces Oxidative Stress and Alters the Redox Balance in Breast Cancer Cells

**FIGURE 5:**
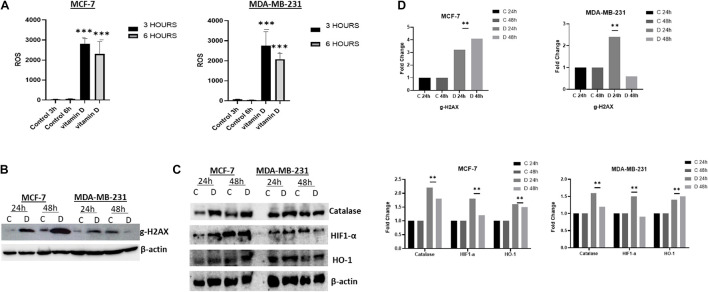
Vitamin D alters the redox balance in BRCA cells in favor of oxidative stress. **(A)** ROS level expression following 3 and 6 h post vitamin D treatment. Expression of **(B)** the DNA damage sensor, g-H2AX, and **(C)** the redox balance regulators proteins catalase, Hif-1α and OH-1 levels in MCF-7 and MDA-MB-231 cells following at 24 and 48 h post-treatment with vitamin D. The data shown are representative of three separate experiments. **(D)** Quantitative analysis of relative protein band density after normalization to β-actin and compared to the control. **Represents statistically significant change (*P* < 0.01) and ***Represents statistically significant change (*P* < 0.001).

The observation that vitamin D disrupts cellular iron homeostasis raised the possibility that this could consequently influence the oxidative stress status in treated cells. To address this issue, we examined the ROS level in vitamin D-treated MCF-7 and MDA-MB-231 cells as shown in Figure 5A which indicate a significant increase in both cell lines post 3 and 6 h vitamin D treatment. Furthermore, we confirmed redox balance disturbance by assaying for the expression of several stress-related markers including catalase, γ-H2AX, HIF-1α, and HO-1. A significant increase of γ-H2AX was observed in MCF-7 treated at 24 and 48 h and in MDA-MB-231 treated at 24 h (Figure 5B, top panel). Likewise, catalase activity in vitamin D-treated MCF-7 cells at 24 and 48 h and in MDA-MB-231 cells at 24 h was significantly increased (Figures 5C,D, first panel from top). Compared to the control, HIF-1α was highly increased in MCF-7 and MDA-MB-231 (Figure 5C, second panel from top). The expression level of Heme Oxygenase 1 (HO-1) protein was significantly upregulated in both cell lines at both time points following vitamin D treatment (Figure 5C, third panel from top).

**FIGURE 6:**
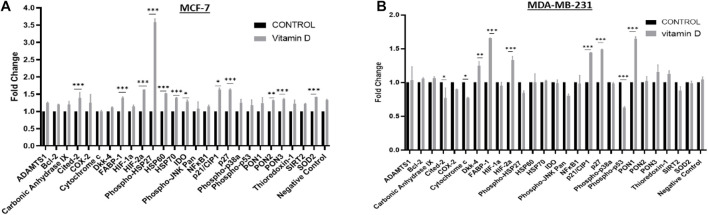
Proteome profiling of cell stress regulators in vitamin D-treated BRCA cells. Expression status of key cell stress regulators in **(A)** MCF-7 and **(B)** MDA-MB-231 cells using cell lysates prepared from cells at 24 h post-treatment with vitamin D. *Denotes the presence of statistically significant changes (*p* < 0.05) in protein expression in treated vs. control samples. **Represents statistically significant change (*P* < 0.01), ***Represents statistically significant change (*P* < 0.001).

Oxidative stress/redox balance was further investigated in treated cells, the proteome profiler for human cell stress markers was employed. In Figure 6A, MCF-7 cells treated at 24 h show that there is a significant increase in phosphor-HSP27, Cited-2, FABP-1, HIF-2α, HSP60, HSP70, p27, PON3, and SOD2. A significant increase in FABP-1, HIF-2α, p21/CIP-1, p27, and PON1 stress markers was observed in MDA-MB-231 at 24 h after treatment with vitamin D (Figure 6B).

#### Vitamin D Disrupts Cell-Cycling in MCF-7 and MDA-MB-231 Cells

**FIGURE 7:**
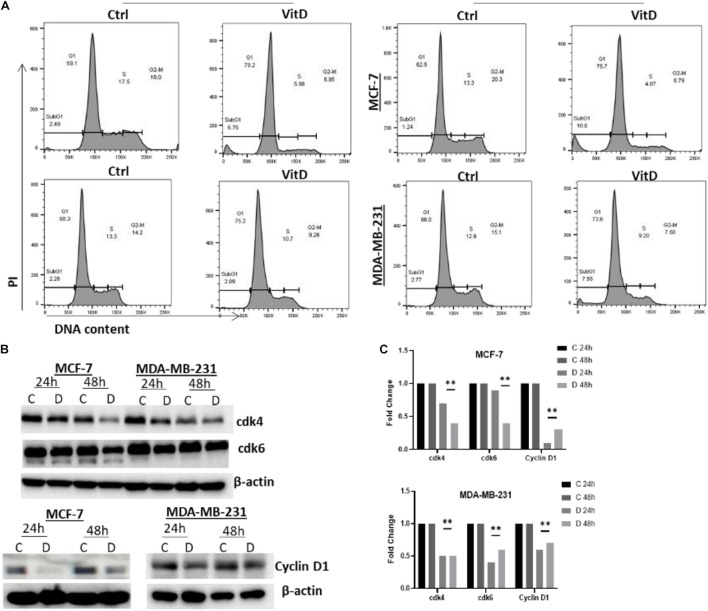
Vitamin D treatment leads to cell cycle arrest at G1/S phase arrests in BRCA cells. **(A)** Cells treated with 10 μM vitamin D for 24 and 48 h as well as PI stain were used for untreated controls and analyzed. **(B)** cdk4 (U/mg protein), cdk6 (U/mg protein), and cyclin D1 (U/mg protein) were assayed for control, and MCF-7 and MDA-MB-231 cells treated with 10 μM of vitamin D for 24 and 48 h. **(C)** Quantitative analysis of relative protein band density after normalization to β-actin and compared to the control. **Represents statistically significant change (*P* < 0.01).

Treatment of vitamin D resulted in a significant disruption in cellular iron metabolism and cell redox balance, we next measured the percentage of cells/cell cycle phases in vitamin D-treated vs. control cells to evaluate the treatment effects on cell cycling. Treatment of MCF-7 with vitamin D for 48 h resulted in a G1/S phase arrest prior to cell cycle analysis (Figure 7). At both 24 and 48 h MCF-7 treated cells, the percentage of G1 phase was higher in relative to untreated controls (79.2% vs. 59.1%) and 48 h (75.7% vs. 62.5%);% of treated vs. untreated MDA-MB-231 at G1 phase was (75.2% vs. 66.3%) at 24 h and (73.6% vs. 66%) at 48 h. Moreover, the expression of cyclin D1 in MCF-7 and MDA-MB-231 at 24 and 48 h post-treatment was significantly downregulated. These findings notwithstanding, a significant decrease in cdk4 and cdk6 in vitamin D-treated MCF-7 and MDA-MB-231 cells at 48 h post-treatment (Figure 7B).

#### Vitamin D Induces Apoptosis and Decreases Cell Growth Potential in Breast Cancer Cells

**FIGURE 8:**
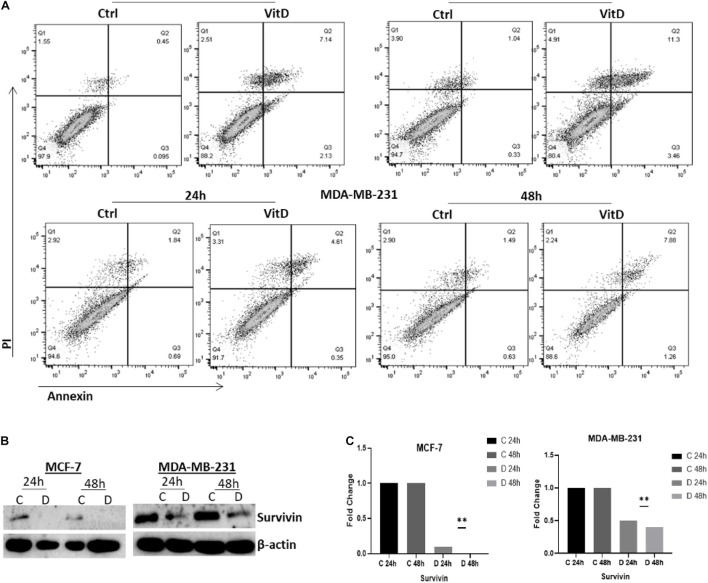
Vitamin D effect on apoptosis and cell survival in BRCA cells. **(A)** The Annexin V/PI flow cytometry-based method used to assess the percentage of apoptotic and pro-apoptotic in MCF-7 and MDA-MB-231 cells treated with 10 μM of vitamin D for 24 or 48 h. **(B)** Survivin expression was assessed in 10 μM of vitamin D-treated cell lysates and from untreated controls at 24 and 48 h post-culture. **(C)** Quantitative analysis of relative protein band density after normalization to β-actin and compared to the control. **Represents statistically significant change (*P* < 0.01).

Annexin V/PI flow cytometry-based method was used to assess the rate of cells growth; percentage of apoptotic cells MCF-7 and MDA-MB-231 treated with vitamin D was significantly increased relative to controls, especially at 48 h where it was 11.3% vs. 1.04% in MCF-7 cells and 7.88% vs. 1.49% in MDA-MB-231 (Figure 8A). Moreover, the expression levels of antiapoptotic protein surviving were assessed in cell lysates of both vitamin D-treated cells and untreated controls by western blotting. Survivin expression showed a significant reduction in MCF-7 and MDA-MB-231 treated cells at 24 and 48 h (Figure 8B).

## Discussion

Data presented here clearly show that vitamin D precipitates significant anti-cancer effects in BRCA cells. This effect is based on disruption of cellular iron metabolism, increasing in oxidative stress, induction of cell cycle arrest, enhancement of apoptosis, and reduction in cell growth potential in two distinct BRCA cell lines, MCF-7 and MDA-MB-231. Our data showed that the expression of key IRGs was modulated after vitamin D by causing a disruption in cellular iron metabolism, vis-à-vis reducing hepcidin synthesis, increasing HIF-1α and FPN expression, and decreasing TFR1 expression. Moreover, vitamin D treatment resulted in a transient LIP and FT depletion. Collectively, this suggests that vitamin D treatment induces an iron release phenotype in BRCA cells. The expression of high HIF-1α protein after vitamin D treatment is in positive agreement with previously published data (Jiang et al., 2010). Moreover, reduction of hepcidin synthesis and increasing of HIF-1α expression is in agreement with the other findings. HIF-1α can negatively regulate the synthesis of hepcidin to enhance cellular iron export to increase systemic demand for iron (Nemeth et al., 2004). A previous study observed that vitamin D binding to the vitamin D receptor reduces hepcidin mRNA expression levels in PBMC monocytes and this is consistent with our finding of vitamin D ability to reduce hepcidin synthesis, THP1 cells, and HepG2 cells (Bacchetta et al., 2014). Proinflammatory cytokines and hepcidin were reported to be significantly downregulated in vitamin D THP-1 treated cells (Zughaier et al., 2014) and to significantly reduce plasma hepcidin concentrations in healthy adults (Smith et al., 2017). Together, these findings are consistent with our results of vitamin D treated breast cancer cells have low hepcidin expression (Figure 3).

Our data showed that reduced hepcidin synthesis paralleled a significant increase in FPN expression. This is consistent with the negative impact of hepcidin on FPN expression and the fact that reduced hepcidin synthesis maintains FPN integrity (Zughaier et al., 2014). Given that TfR1 is a key player in iron import to cells (Wan et al., 2019), suppressed TfR1 expression following vitamin D treatment would likely result in reduced cellular iron content or LIP. Collectively, therefore, reduced hepcidin and TfR1 expression along with increased FPN expression are all consistent with an iron release phenotype as evidenced by decreased LIP content (ΔMFI < 0).

Interestingly and perhaps surprisingly, vitamin D treatment disrupted the redox balance as demonstrated by the high levels of ROS and the increased expression of stress-related markers like γ-H2AX, HIF-1α, and HO-1. This is indicative that vitamin D treatment decreased cellular LIP content, which would be expected to reduce rather than increase oxidative stress. Increasing oxidative stress in treated cells was confirmed by the overexpression of the antioxidant HO-1 (Abo El-Magd and Eraky, 2020) and increased expression of HIF-1α. The ability of vitamin D to inhibit cell growth in BRCA cells by generating ROS and causing DNA damage was observed in previous research (Marchionatti et al., 2009; Bohl et al., 2017). Our findings are consistent with the ability of vitamin D-treated cells to induce DNA disruption as proven by the transitory increased expression of γ-H2AX (Figure 6).

Proteome profiling, which further probed the oxidative stress status in vitamin D-treated cells and which revealed overexpression of cell cycle modulators p21 and p2, may help explain suppressed BRCA growth and proliferation following vitamin treatment. Previous work has reported that calcitriol increases the levels of p21WAF 1/Cip1 and p27Kip1 cell cycle regulators which inhibits the ability of cancer cells to proliferate (Gartel and Tyner, 2002; Audo et al., 2003). Our results also demonstrated a significant increase in catalase expression in vitamin D-treated BRCA. Given that the catalase enzyme is an antioxidant that catalyzes hydrogen peroxide neutralization, its increase further suggests that vitamin D treatment puts cells under significant oxidative stress which triggers antioxidant systems like catalase as means of oxidative stress and cellular damage. It is worth noting here that high metabolic rates in tumor cells associate with increased production of ROS and oxidative stress (Marklund et al., 1982; Sandstrom and Buttke, 1993; Glorieux and Calderon, 2017). Therefore, the ability of vitamin D to target and capitalize on this precarious pathway in cancer cells merits further examination as a potential therapeutic target. In this context, our data showed that vitamin D induced G1/S phase cell cycle arrest associating with downregulated cdk4, cdk6, and cyclin D1 expression (Figure 7) and induced apoptosis which is associated with reduced expression of survivin (Figure 8). These findings are consistent with previous research that further founded that vitamin D supplementation inhibited cell proliferation throughout multipotent mesenchymal cells and arresting cell cycling (Artaza et al., 2010). Additionally, previously published data have suggested that vitamin D induced apoptosis in different types of cancers including breast cancer, colon cancer, and glioma cell lines (Welsh, 1994; Sergeev, 2014). Increasing evidence suggests an important antiproliferative and apoptotic effects of vitamin D in cancer. Importantly, however, new treatment strategies are needed to reverse the multidrug resistance in many cancer types by using vitamin D in combination with different drugs and investigating the related pathways.

## Conclusion

**FIGURE 9:**
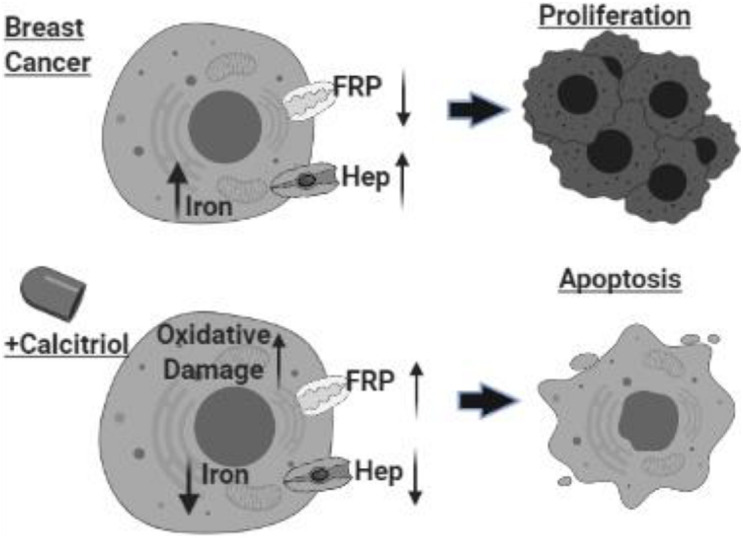
Schematic illustration of study conclusion. Created with BioRender.com.

In conclusion, as shown in Figure 9, our findings highlighted the complex relationship between vitamin D and cellular iron metabolism suggesting that vitamin D could have significant anti-cancer effects by disrupting cellular iron homeostasis. The molecular pathway of vitamin D ability to alter the expression of key IRGs requires additional conformational work.

## Data Availability Statement

The original contributions presented in the study are included in the article/supplementary material, further inquiries can be directed to the corresponding author/s.

## Author Contributions

KB and MH: conceptualization and funding acquisition. KB, LS, JS, AA-A, AK, JM, and SG: visualization, methodology, and validation. JS: software. KB and LS: formal analysis. KB, LS, JS, AA-A, AK, and JM: investigation. KB, LS, JS, AA-A, and JM: data curation. KB, LS, and MH: writing—original draft preparation. KB, LS, and MH: writing—review and editing. KB: supervision and project administration. All authors have read and agreed to the published version of the manuscript.

## Conflict of Interest

The authors declare that the research was conducted in the absence of any commercial or financial relationships that could be construed as a potential conflict of interest.

## Publisher’s Note

All claims expressed in this article are solely those of the authors and do not necessarily represent those of their affiliated organizations, or those of the publisher, the editors and the reviewers. Any product that may be evaluated in this article, or claim that may be made by its manufacturer, is not guaranteed or endorsed by the publisher.

## Footnotes

1
https://www.ncbi.nlm.nih.gov/geo/
